# The effects of different gait patterns on knee joint biomechanics and dynamic stability during stair ascent in healthy adults

**DOI:** 10.3389/fspor.2026.1924813

**Published:** 2026-09-30

**Authors:** Gangzheng Yi, Lian Duan, Yang Sun, Yuan Gao, Dapeng Wang

**Affiliations:** 1 School of Physical Education, Yanshan University, Qinhuangdao, Hebei, China; 2 China Volleyball College, Beijing Sport University, Beijing, China; 3 School of Physical Education, Sanya University, Sanya, Hainan, China

**Keywords:** dynamic stability, gait patterns, healthy adults, knee biomechanics, stair ascent

## Abstract

**Objective:**

This study aimed to investigate the effects of different stair-ascent gait patterns on knee joint biomechanics and dynamic postural stability.

**Methods:**

This cross-sectional, repeated-measures laboratory study was approved by the Ethics Committee of Qinhuangdao First Hospital [approval no. (2025K-135-01)]. Experimental procedures were conducted at Qinhuangdao First Hospital, and data analysis was performed at Yanshan University, which served as the collaborating institution for study design and statistical processing. Participants were recruited between 03 December 2024 and 18 December 2024. Fourteen healthy young males (mean age: 19.8 years; mean body mass: 72.2 kg) were enrolled and instructed to ascend stairs using two distinct gait patterns: a regular step-by-step (alternating) pattern, in which one step height was ascended per gait cycle, and a double-step (skip-step) pattern, in which two step heights were ascended per cycle by skipping one intervening step. Kinematic and kinetic data were synchronously recorded using a Qualisys infrared motion-capture system and a Kistler force platform. As each participant performed both conditions, all statistical comparisons were conducted using paired-sample *t*-tests. Dynamic stability and knee loading were quantified by the margin of stability (MOS) and patellofemoral joint stress (PFJS), respectively.

**Results:**

Throughout the gait cycle, no significant difference was observed MOSml between the two gait patterns (*P* = 0.318). In the anterior-posterior (AP) direction, however, the MOS for both patterns remained negative and decreased over time, with the double-step pattern yielding significantly lower MOSap values compared with the regular step-by-step pattern (*P* = 0.002). At the instant of left foot-off, significant differences were detected between patterns in right knee joint angle, right knee joint moment, net joint moment, effective quadriceps muscle lever arm, quadriceps force (QF), angle between the quadriceps tendon and patellar ligament, patellofemoral joint force (PFJF), PFJS, and patellofemoral contact area (all *P* < 0.001).

**Conclusions:**

In healthy young males, the double-step stair-ascent pattern was associated with reduced anterior-posterior dynamic stability, reflecting a decreased anterior-posterior stability margin. This pattern was also associated with elevated PFJS, reflecting greater compressive loading of the knee joint. Because of the cross-sectional design, these findings represent observed associations rather than causal mechanisms. Accordingly, lower-limb strengthening exercises and modifications to stair-climbing strategy may constitute reasonable hypotheses for mitigating stability decline and mechanical loading; however, these should be tested in future studies before being translated into clinical recommendations.

## 1 Introduction

Stair walking constitutes a frequent functional activity in daily life and is also recognized as an efficient form of physical exercise. Compared with level walking, stair ascent has been shown to confer greater benefits in enhancing lower-limb muscle strength, improving cardiorespiratory fitness, controlling body fat, and reducing postprandial hyperglycemia [see (1)]. However, stair negotiation is also associated with a substantially greater decline in stability. Relative to level walking, it typically involves increased postural instability, a decline in stability, and distinct lower-limb biomechanical profiles. Consequently, stair ascent imposes greater demands on postural control, requiring larger support moments at the lower-limb joints and greater knee muscle strength [see (2)].

Among healthy individuals, two primary gait habits are commonly observed during stair ascent: the regular step-by-step (alternating) pattern and the double-step (skip-step) pattern. In the regular pattern, the body maintains center-of-mass (COM) stability through more frequent lower-limb alternations, with the knee and ankle joints performing smaller-amplitude flexion–extension and shock-absorbing movements [see (3)]. In contrast, the double-step pattern necessitates greater explosive muscle power to propel the body over a larger vertical distance, resulting in higher instantaneous loads on the hip and knee joints, accompanied by a significantly increased trunk lean angle and COM displacement range. Previous studies have documented differences in kinematic and kinetic parameters between these two strategies; however, most analyses have remained at the level of static comparisons of external mechanical variables, without examining the intrinsic relationship between postural stability maintenance and joint structural loading under different strategies. Dynamic stability reflects the real-time capacity of the neuromuscular system to counteract internal and external perturbations, whereas PFJS is directly associated with the risk of joint degeneration. These two aspects may be mutually constraining during gait transitions, yet prior research has tended to address them separately, lacking quantitative analyses within a unified framework.

Furthermore, different gait strategies not only alter lower-limb mechanical performance but also involve deeper neuromuscular control mechanisms. Recent evidence indicates that stair locomotion is not merely a mechanical output of the musculoskeletal system but rather a complex process of neuromuscular coordination. For instance, in a longitudinal study of patients with anterior cruciate ligament reconstruction (ACLR), Wei et al. [see (4)] (2024) found that participants compensated for post-injury deficits by reconstructing motor modules during stair tasks, revealing the dynamic adaptive logic of the nervous system in response to changing task demands. These clinical findings offer important references for understanding motor control strategies during stair walking, suggesting that even in healthy populations, the human body may adopt specific neuromuscular coordination patterns to maintain balance when confronted with different mechanical loads between the regular and double-step modes.

Despite these advances, current research on stair ascent has largely concentrated on overall movement characteristics or on movement optimisation in specific pathological populations [see (5, 19)], whereas systematic comparative analyses of biomechanical differences and the underlying stability regulation mechanisms in healthy adults under different fundamental gait patterns remain insufficient. Therefore, the present study was designed to compare dynamic stability and patellofemoral joint mechanics between the regular step-by-step and double-step stair-ascent modes. This investigation aims not only to delineate the effects of different gait patterns on postural control but also to enrich the theoretical framework for preventing stability decline during stair walking by comparing performances under varying mechanical demands, and to provide a biomechanical baseline for subsequent movement optimisation.

Based on the foregoing analysis, the following hypothesis was formulated: in healthy adults, the double-step pattern during stair ascent, relative to the regular step-by-step pattern, is associated with reduced dynamic stability and elevated PFJS.

## 2 Participants and methods

### 2.1 Participants

Sample size estimation was performed using G*Power software for a paired-samples *t*-test [see (4)]. With Cohen's d-z set at 0.8 (consistent with the expected effect size), a Type I error probability (*α*) of 0.05, and a statistical power (1−*β*) of 0.80, the minimum required sample size was calculated as 11 participants. To account for an anticipated missing-data rate of 30% in motion-capture experiments, the target enrollment was adjusted to 14 participants. Accordingly, 14 healthy young males were ultimately included in the study. Their basic demographic characteristics are summarised below (Table 1).

**Table 1:** Basic information of participants (*N* = 14).

Age (years)	Height (cm)	Body Mass (kg)	Body Mass Index (kg/m^2^)	Dominant Leg
19.75 ± 0.86	181.24 ± 4.62	72.21 ± 7.66	22.06 ± 2.94	right-leg

**Table 2:** Comparison of biomechanical indicators between regular step-by-step mode and double-step mode.

Stage	Label	Regular-Step	Double-Step	MD (95% CI)	t	P	Cohen's d
RFS	KA	58.4 ± 5.8	65.8 ± 5.7	7.3(−10.7,−4.1)	−4.841	0.001	1.343
	KM	−0.080 ± 0.052	−0.08 ± 0.068	0.0007(−0.04,0.04)	0.04	0.985	0.011
	Mnet	−0.78 ± 0.51	−0.79 ± 0.67	0.007(−0.4,0.4)	0.04	0.985	0.011
	QMA	2.9 ± 0.28	2.54 ± 0.21	0.32 (0.2,0.5)	4.733	0.001	1.313
	Fq	−27 ± 17	−31 ± 26	3.8(−11.2,18.8)	0.553	0.590	0.153
	*β*	61 ± 3.05	65 ± 3.04	3.9(−5.7,−2,2)	−4.841	0.001	−1.343
	FPF	−27 ± 17	−34 ± 29	5.8(−10.3,21.9)	0.781	0.450	0.217
	SPFCA	460 ± 58	537 ± 62	76.8(−109.7,−43.8)	−5.075	0.001	−1.408
	PFJS	−0.06 ± 0.04	−0.06 ± 0.05	0.002(−0.03,0.03)	0.119	0.907	0.033
LOFF	KA	45.3 ± 5.2	64.9 ± 6.9	19.6(−23.5,−15.7)	−10.896	0.001	−3.022
	KM	0.9 ± 0.26	1.3 ± 0.24	0.4(−0.5,−0.3)	−6.435	0.001	−1.785
	Mnet	9.0 ± 2.5	13 ± 2.4	4.0(−5.3, −2.6)	−6.435	0.001	−1.785
	QMA	3.5 ± .23	2.6 ± .27	0.9 (0.7, 1.0)	11.466	0.001	3.18
	Fq	243 ± 49	507 ± 123	240.9(−295.1, −186.7)	−9.682	0.001	−2.685
	β	54.5 ± 2.8	65 ± 3.7	10.3(−12.5,−8.3)	−10.896	0.001	−3.022
	FPF	246 ± 109	548 ± 157	301.0(−367.6,−234.4)	−9.85	0.001	−2.732
	SPFCA	344 ± 43	528 ± 75	183.4(−222.6,−144.3)	−10.122	0.001	−2.832
	PFJS	0.70 ± 0.19	1.02 ± .19	0.3(−0.4,−0.2)	−6.982	0.001	−1.936
LFS	KA	3.8 ± 6.0	11.8 ± 8.9	8.0(−13.0,−2.8)	−3.396	0.009	−0.942
	KM	−0.6 ± .19	−0.1 ± 0.36	0.5(−0.6,−0.3)	−5.33	0.001	−1.478
	Mnet	−0.1 ± 1.85	−1.4 ± 3.5	1.3(−5.9,−2.5)	−5.33	0.001	−1.478
	QMA	3.1 ± .21	3.4 ± .31	0.3(−0.5,−0.1)	−3.415	0.005	−0.947
	Fq	−180 ± 67	−45 ± 96	135(−183.5,−86.1)	−6.028	0.001	−1.672
	β	32.9 ± 3.2	37.1 ± 4.7	4.2(−6.9,−1.5)	−3.396	0.005	−0.942
	FPF	−98.1 ± 31	−24.5 ± 67	74.4(−106.9,−42.0)	−5.001	0.001	−1.387
	SPFCA	158.2 ± 9.3	176.4 ± 26	18.2(−33.4,−3.0)	−2.609	0.037	−0.723
	PFJS	−0.6 ± 0.21	−0.1 ± 0.33	0.5(−0.6,−0.3)	−6.171	0.001	−1.711
ROFF	KA	16.2 ± 5.9	6.08 ± 6.43	10.1 (6.1,14.2)	3.043	0.001	1.502
	KM	−0.22 ± 0.05	−0.18 ± 0.022	0.03(−0.07,0.00)	−2.214	0.078	−0.598
	Mnet	−2.15 ± 0.52	−1.81 ± 0.21	0.3(−0.7,0.004)	−2.214	0.078	−0.598
	QMA	3.58 ± 0.22	3.21 ± 0.23	0.4 (0.2,0.5)	3.043	0.001	1.502
	Fq	−60 ± 15.9	−56.1 ± 7.23	3.6(−14.0,6.9)	−1.065	0.556	−0.21
	β	39.0 ± 3.1	33.6 ± 3.4	5.4 (3.2,7.5)	3.043	0.001	1.502
	FPF	−40 ± 9.64	−32.6 ± 4.1	7.3(−13.5,−1.2)	−2.543	0.023	−0.724
	SPFCA	186 ± 20.2	161 ± 12.4	24.1 (11.7,36.5)	2.587	0.001	1.177
	PFJS	−0.21 ± 0.059	−0.2 ± 0.024	0.02(−0.05,0.02)	−1.226	0.507	−0.244

*P*-values were adjusted for multiple comparisons using the Benjamini–Hochberg false discovery rate method (m = 60).

Inclusion criteria were as follows: (1) no history of neuromusculoskeletal diseases or cognitive impairment, with corrected binocular visual acuity of 1.0 or higher; (2) no significant lower-limb joint injury within the preceding six months; (3) no vigorous exercise performed within 24 h prior to the experiment; and (4) right-leg dominance. Furthermore, all participants were able to understand the experimental procedures and provided written informed consent.

Exclusion criteria were as follows: (1) any gait abnormalities or balance disorders resulting from neurological or musculoskeletal injuries; and (2) a history of lower-limb fractures. All participants volunteered to take part and provided written informed consent prior to the experiment.

### 2.2 Methods

#### 2.2.1 Instrumentation

Kinematic data were acquired and preprocessed using a Qualisys three-dimensional (3D) motion capture system (Qualisys, Sweden) comprising eight high-speed cameras (Arqus series), with a sampling frequency of 100 Hz. Ground reaction forces were recorded using four Kistler 3D force plates (Model 9260AA6, Kistler Instruments, Switzerland), which were embedded into the first, second, third, and fourth steps of a custom-built staircase (Figure 1) and sampled at 2000Hz. Synchronisation between the force plates and the motion capture system was achieved via an analogue-to-digital converter. Retro-reflective markers, adhesive tape, and kinesiology tape were also used as auxiliary materials.

**Figure 1:**
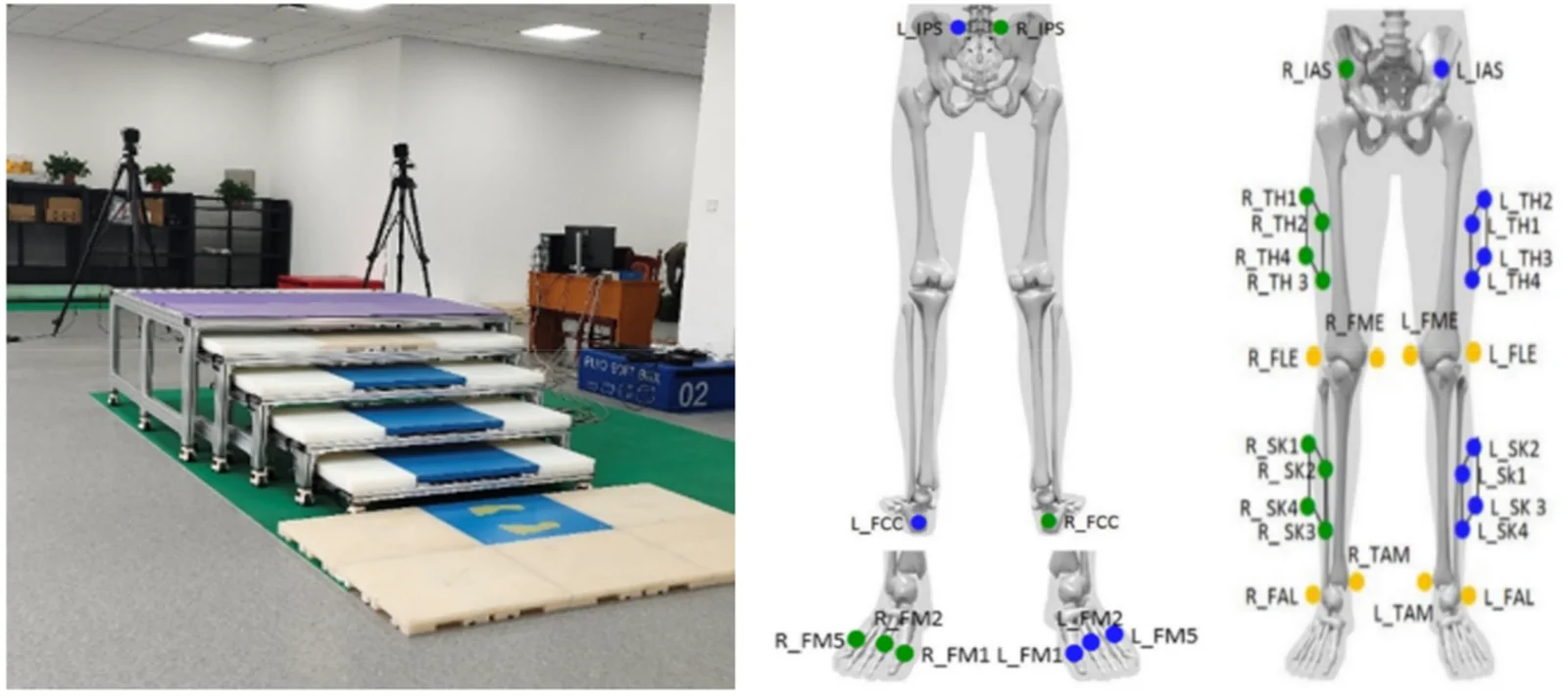
Schematic diagram of the simulated staircase and lower-body marker point placements.

#### 2.2.2 Testing protocol

Prior to the formal experiment, participants were required to change into tight-fitting clothing and footwear provided by the laboratory. Subsequently, anthropometric data were collected, and the dominant leg was determined using a ball-kicking test [see (6)] all participants were confirmed to have a right-dominant leg. Twenty retro-reflective markers (Figure 1) were then placed on anatomical landmarks of the trunk, pelvis, thighs, shanks, and feet according to the Visual3D model. Before testing, each participant completed a 5-minute warm-up and was given time to familiarise themselves with the experimental environment.

The testing order was standardised, with the regular step-by-step condition always performed first, followed by the double-step condition. This arrangement was primarily adopted to allow participants to habituate to the laboratory setting, the motion-capture system, and the overall experimental pace before being exposed to the spatially perturbed double-step paradigm, which slightly increases the risk of tripping or balance loss. However, it is important to acknowledge that this fixed order may introduce potential order effects, including learning, fatigue, or habituation, which could influence the biomechanical outcomes. Although such effects cannot be entirely ruled out, their possible impact on the comparison between conditions is considered in the interpretation of the results (see Discussion/Limitations).

At the beginning of each session, a practice trial was conducted. Thereafter, the participant stood 15 cm from the edge of the first step and awaited the command. Upon a verbal “Start” signal, they ascended the staircase with the right foot first. A metronome was used to maintain a uniform cadence of 120 beats per minute (BPM), and participants followed the metronome cues to complete the stair-climbing movement until a single-leg stance on the left foot was achieved, marking the end of the first trial. Although the cadence was fixed, it is acknowledged that actual forward velocity is not strictly determined by cadence alone; therefore, the potential confounding effect of velocity on biomechanical outcomes has been addressed in the interpretation of the results (see Discussion/Limitations). The participant then returned to the starting position, and the test was repeated to obtain three valid trials for each condition.

Two distinct gait tasks were defined:(1) Regular step-by-step (alternating) pattern: the swing leg lands on the immediately adjacent upper step within one swing phase, with a single-step elevation per gait cycle, representing conventional physiological stair climbing with alternating sequential support (Figure 2). (2) Double-step (skip-step) pattern: the swing leg crosses two consecutive steps within a single swing phase and lands on the second upper step, achieving a double-step height elevation. Continuous alternating foot support was required throughout the trial, and airborne jumping was prohibited (Figure 3).

**Figure 2:**
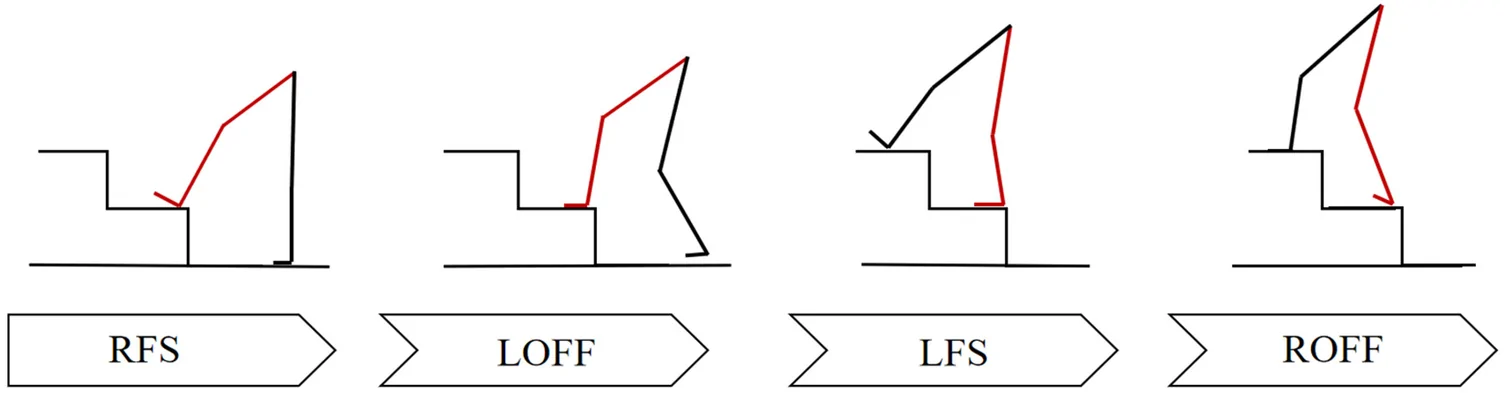
Schematic diagram of the regular step-by-step (alternating) pattern.

**Figure 3:**
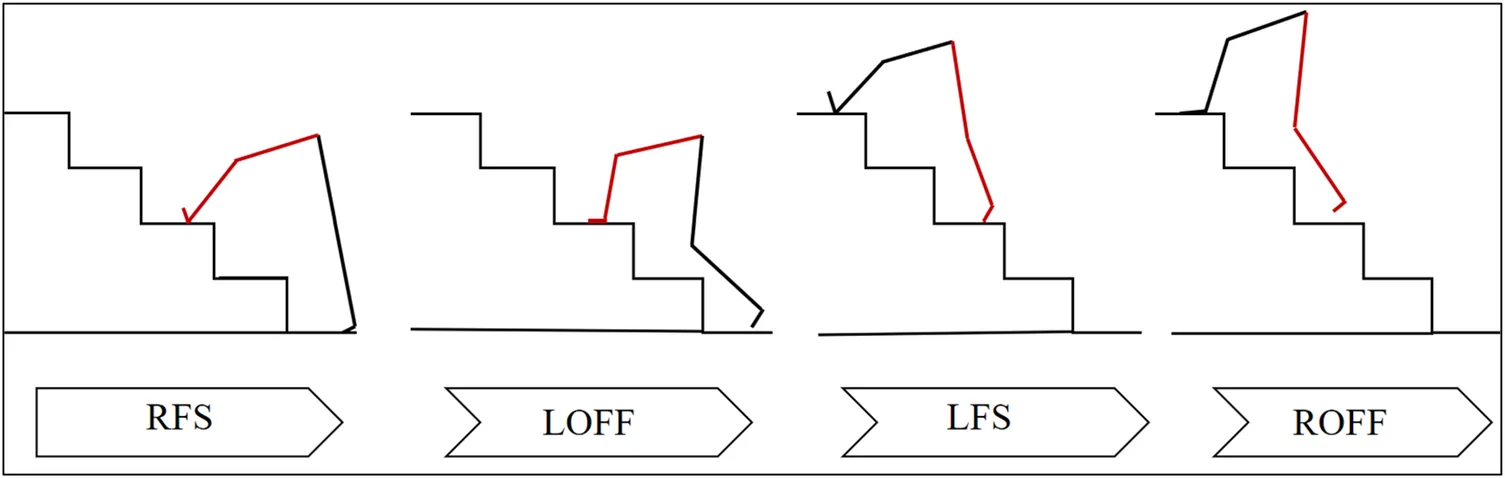
Schematic diagram of the double-step (skip-step) pattern.

### 2.3 Data processing

Paired-samples t-tests were conducted to examine the effects of stair-ascent gait patterns on basic gait cycle characteristics and joint range of motion. Prior to performing the paired t-tests, the normality of the data was assessed using the Shapiro–Wilk test. To account for multiple comparisons, the Benjamini–Hochberg procedure was applied to control the false discovery rate across all 60 paired comparisons, and adjusted *p*-values are reported. The stance phase of the dominant leg was extracted for dynamic stability analysis. All data were normalised to 100% of the gait cycle using OriginPro 2024 software. For each participant, the data from the three valid trials were averaged prior to comparative analysis.

Based on the findings of Bossé et al [see (7)], who identified transitions between different support phases as the most unstable moments during stair negotiation, dynamic stability was analysed at four critical events:(1) Right Foot Strike (RFS): transition from left single-leg support to double support;(2) Left Foot Off (LOFF): transition from double support to right single-leg support;(3) Left Foot Strike (LFS): transition from right single-leg support to double support;(4) Right Foot Off (ROFF): transition from double support to left single-leg support.

Foot strike was defined as the instant at which the vertical ground reaction force (vGRF) recorded by the force plate reached or exceeded 20 N [see (8, 20)].

### 2.4 Testing indicators

#### 2.4.1 Dynamic stability

Hof et al. (9) found that, in gait tests, the moment when the swing leg contacts the ground is the most unstable moment for the human body, at which time the dynamic stability margin is the smallest. Therefore, this study selects the moment when the non-dominant foot contacts the ground for the dynamic stability margin analysis. The calculation formula of the dynamic stability margin is as follows (9):

$$\omega_{0}=\sqrt{\left(g/l\right)}$$ (1)

$$CM=COM+VCOM/\omega_{0}$$ (2)

MOS=Bmax−CM (3)

Figure 4 here, *ω*₀ is the frequency of the human inverted pendulum model, g is the gravitational acceleration, and l is the vertical distance from the body's center of mass (CoM) to the ground; CM is the extrapolated center of mass, CoM denotes the displacement of the center of mass at a given moment, and VCoM denotes the velocity of the center of mass at a given moment; MoS is the dynamic stability margin at a given moment, and Bmax is the maximum value of the support surface boundary in a given direction. In this study, Bmax is represented by the position of the center of pressure (CoP) of the foot.

**Figure 4:**
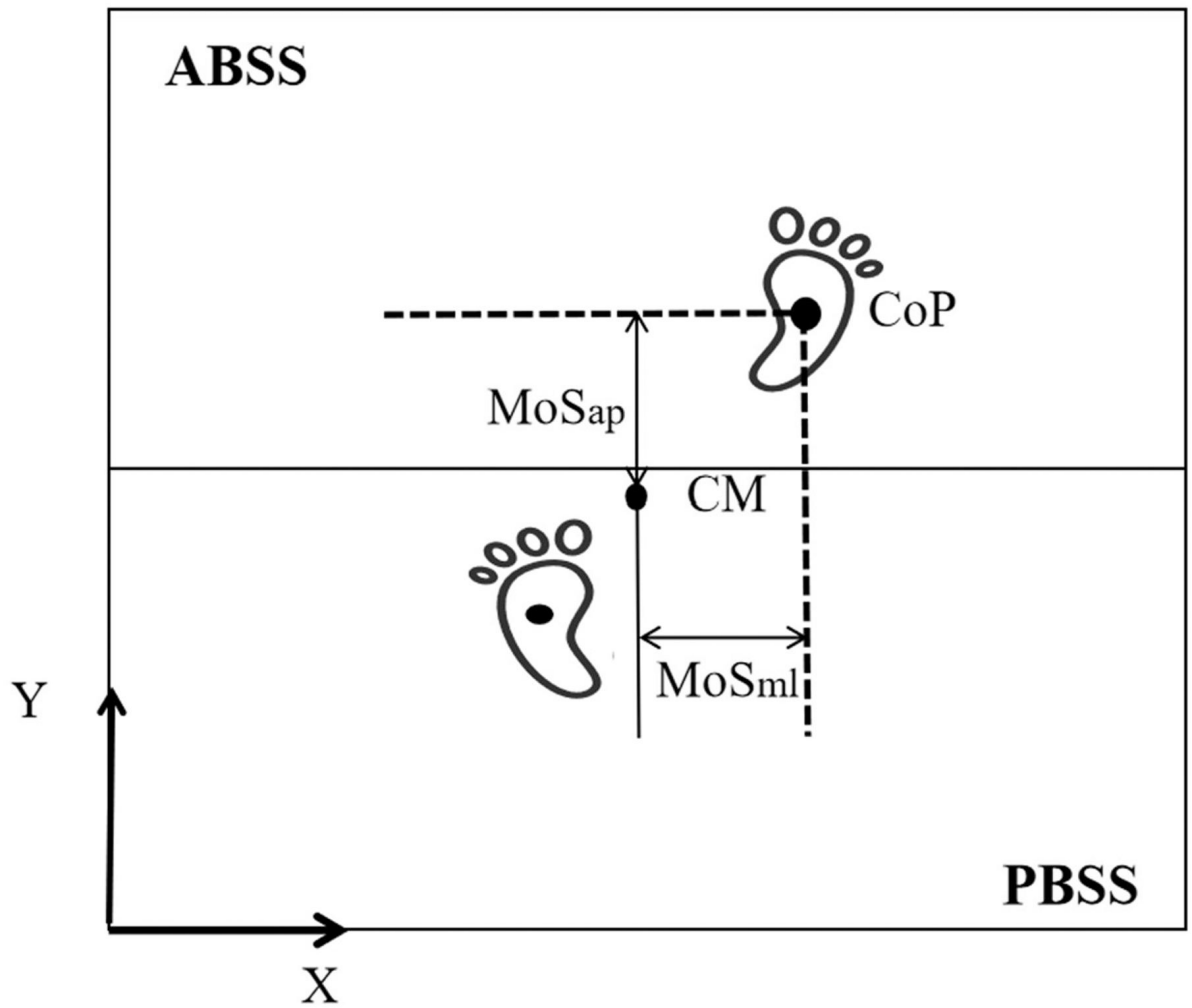
Schematic diagram of dynamic stability. ABSS, anterior boundary of the support surface; PBSS, posterior boundary of the support surface.

To validate the applicability of this COP-based functional boundary specifically for stair ascent, it is worth noting that the COM-COP separation during stair negotiation has been employed as a sensitive indicator of dynamic postural stability in previous empirical studies. Mian et al [see (10)] compared the COM motion and its separation from the COP in young and older men during stair ascent and descent, demonstrating that frontal-plane dynamic stability during stair negotiation can be effectively captured through COP-related measures. Reeves et al [see (11)] further confirmed that older adults exhibit reduced COM-COP separation in the frontal plane during stair ascent, reflecting a more cautious control strategy. More recently, Dussault-Picard et al [see (12)] systematically compared marker-based and COP-based methods for computing the MoS and reported that the COP-based approach, as originally recommended by Hof's model, yields improved accuracy in defining the effective stability boundary. Additionally, Millard and Sloot [(see (13)] introduced a functional base of support (fBOS) polygon model and demonstrated that using the functional COP-excursion boundary instead of the marker-based BOS improves MoS accuracy by 20% of foot width and 16% of foot length (22). Although most of these validation studies were conducted during level walking or standing, the fundamental mechanical rationale-that COP defines the physiologically available limit for corrective torque generation-is task-independent and thus equally applicable to stair ascent. We acknowledge that direct validation of the Bmax-CM formula specifically for stair ascent remains relatively limited, and we suggest that future studies establish its test-retest reliability and predictive validity during stair negotiation.

Accordingly, in this study we replace the physical BOS boundary with the COP-defined functional stability boundary (denoted as Bmax) in the stability calculation. MOS at a given instant is then computed as:

MOS = Bmax - CM

where CM = COM + VCOM/*ω*₀ is the extrapolated centre of mass position, VCOM is the COM velocity, and *ω*₀ =  $\sqrt{\left(g/l\right)}$ is the natural frequency of the inverted pendulum (g = gravity, l = vertical distance from COM to ground). It is important to note that this MOS represents a modified stability metric based on the functional (COP-defined) boundary, rather than the conventional MOS defined with respect to the physical edges of the base of support (21). This formula directly quantifies the instantaneous distance between the extrapolated COM and the functional boundary. A positive MOS indicates that CM lies within the COP-defined support boundary, meaning the current COM state can be stabilised by modulating COP within its physiological range; conversely, a negative MOS indicates that CM has exceeded this functional boundary, implying that the controller cannot generate sufficient corrective torque with the current foot placement, and that stepping or other compensatory actions are required to prevent stability decline (23).

In the sagittal plane, the anterior direction is defined as positive, and the Bmax used in Equation (3) specifically refers to the anterior functional stability boundary. Under this coordinate definition, a positive MOS indicates that the CM is positioned posterior to this anterior COP boundary (i.e., the COM state remains within the functional stability margin), whereas a negative MOS indicates that the CM has exceeded this anterior boundary, reflecting an increased risk of forward instability. Since this MOS metric is referenced strictly to the anterior boundary, it does not assess backward fall risk unless the posterior boundary is separately defined and analyzed using a reversed sign convention. In the coronal plane, a positive value indicates outward velocity, with the CM positioned medial to the COP; negative values indicate the opposite conditions. Within this defined coordinate framework, a larger positive MOS reflects a greater stability margin in the corresponding direction, while a negative MOS indicates instability, with more negative values representing greater stability decline in that specific direction.

#### 2.4.2 Patellofemoral joint stress

Calculation of Patellofemoral Joint Mechanics:

The biomechanical indices of the patellofemoral joint in this study were calculated based on the models proposed by Bresnahan [see (14)] and Vannatta [see (15)]. The specific calculations are as follows:

Calculation of QF:

$$LA=\left(\begin{array}{c} \begin{array}{c} 0.036\theta +3.0(0\leq \theta <30)\\ -0.043\theta +5.4(30\leq \theta <60)\\ -0.027\theta +4.3(60\leq \theta <90) \end{array}\\ 2.09(\theta \geq 90) \end{array}\right)\;$$ (1)

QF [see (14)] was calculated as the ratio of the knee extension moment to the effective quadriceps moment arm. In Equation (1), the Quadriceps Effective Moment Arm (LA) is defined as a piecewise function of the sagittal plane knee angle (θ).

$$M_{EXT}=M_{NET}$$ (2)

$$QF_{\left(\theta i\right)}=M_{EXT(\theta i)}/[LA(\theta_{i})\times 0.01]$$ (3)

In Equation (2), ***MEXT*(*Nm)***(Nm) represents the sagittal plane knee extension moment, and ***MNET*(*Nm)***(Nm) represents the sagittal plane net knee joint moment. In Equation (3), $\theta_{i}$ represents the knee flexion-extension angle at the i-th frame.

Calculation of Patellofemoral Joint Force (PFJF):

β=30.46+0.53θ (4)

$$PFJF=2QF_{sin}(\beta /2)$$ (5)

PFJF was calculated according to Equations (4, 5), and *β* (°) is the angle between the quadriceps muscle force line and the patellar ligament tension line.

The PFJS was calculated as follows [see (14)]:

$$PFCA_{\left(\theta_{i}\right)}=0.078\times \theta_{i}^{2}+0.6763\times \theta_{i}+151.75$$ (6)

$$PFJS_{\left(\theta_{i}\right)}=PFJF_{\left(\theta_{i}\right)}/PFCA_{\left(\theta_{i}\right)}$$ (7)

The Patellofemoral Contact Area (PFCA) is a function of the sagittal plane knee angle (θ) and is calculated using Equations (6, 7). PFJS is defined as the ratio of PFJF to PFCA.

### 2.5 Statistical analysis

The Shapiro–Wilk test was used to examine the normality of the dependent variables using SPSS 27.0. Paired-sample t-tests were employed to statistically analyze indicators including PFJS and dynamic stability. All data are presented as mean ± standard deviation (SD), and a significance level of *P* < 0.05 was considered statistically significant. In addition to statistical significance (defined as a two-tailed *P* < 0.05), the 95% confidence intervals (95% CI) for the mean differences and Cohen's d-z effect sizes were calculated to facilitate interpretation of the practical significance of the findings.

## 3 Results

### 3.1 Differences in patellofemoral joint mechanics between different gait patterns during stair ascent

Figure 5 illustrates the differences in knee biomechanical parameters between double-step and regular step-by-step during stair ascent during stair ascent across four gait instants. Synthesizing the data from all four phases, the most clinically meaningful findings of the double-step pattern are concentrated in the following three aspects.

**Figure 5:**
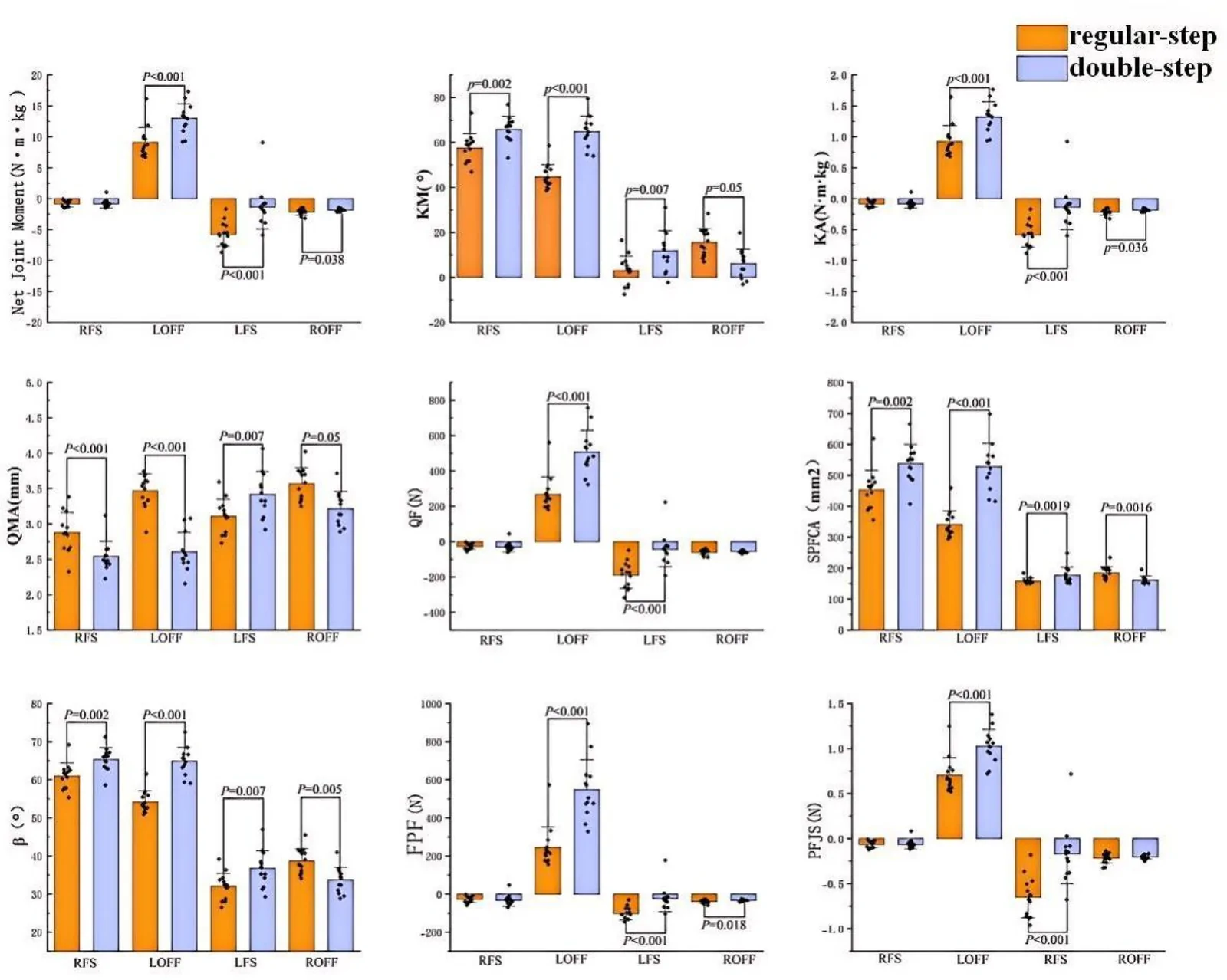
Comparison of biomechanical metrics between regular step-by-step and double-step strategies. KA represents the right knee joint angle; KM represents the right knee joint moment, while Net denotes the net knee moment. QF is the quadriceps muscle force; *β* is the angle between the patellar ligament force and the quadriceps line of action. PFJF is the patellofemoral joint force; QMA is the effective moment arm of the quadriceps; SPFCA is the patellofemoral contact area; and PFJS is the patellofemoral joint stress (force per unit contact area).

First, in terms of joint loading, the double-step pattern exhibited significantly higher PFJS, patellofemoral joint force, and contact area at LOFF and LFS-the critical phases of single-leg support and weight transfer (all *P* < 0.001), indicating that this strategy imposes the greatest compressive demand on the patellofemoral joint during these periods. Notably, the increase in PFJS was most pronounced at LFS, making this instant the clinically most relevant peak-load phase.

Second, regarding muscle efficiency, the double-step pattern demonstrated a significantly smaller effective quadriceps moment arm at all observed instants (RFS and LOFF: *P* < 0.001; LFS: *P* = 0.007; ROFF: *P* = 0.05), while simultaneously generating significantly greater quadriceps muscle force at LOFF and LFS (both *P* < 0.001). This combination of increased force output alongside a shortened moment arm indicates that the double-step pattern relies on a mechanically less advantageous contraction strategy. However, a reduced moment arm and increased force alone do not establish that the contraction is physiologically inefficient, nor do they directly demonstrate higher energy expenditure or an earlier onset of muscle fatigue; these clinical consequences should be regarded as hypotheses requiring direct physiological assessment (e.g., measures of energy cost and muscle fatigue).

Third, in moment output, the double-step pattern produced significantly greater knee joint moments and net joint moments at LOFF and LFS (all *P* < 0.001), and these values remained elevated at ROFF (*P* = 0.036 and *P* = 0.038, respectively). Higher joint moments imply that the knee structures must withstand greater external loads, posing a potential clinical risk of increased joint shear forces and cartilage wear.

Additionally, the knee flexion angle in the double-step pattern was significantly greater than that in the control pattern from RFS through LFS, but became significantly smaller at ROFF (*P* = 0.05). This phase-dependent variation in joint posture may underlie the time-specific changes in the angle between the QF line and the patellar ligament tension (RFS: *P* = 0.002; LOFF: *P* < 0.001; LFS: *P* = 0.007; ROFF: *P* = 0.005). An increase in this angle directly amplifies the compressive component of the patella against the femur, which is a key mechanical mechanism driving the elevation of PFJS.

### 3.2 Differences in dynamic stability between gait patterns during stair ascent

Figure 6 compares the dynamic postural stability profiles between the double-step and the regular step-by-step during stair ascent, as measured by three key indices: CMap, VCOMap, and MOSap. To clarify the functional implications, the findings are synthesized by stability domain below.

**Figure 6:**
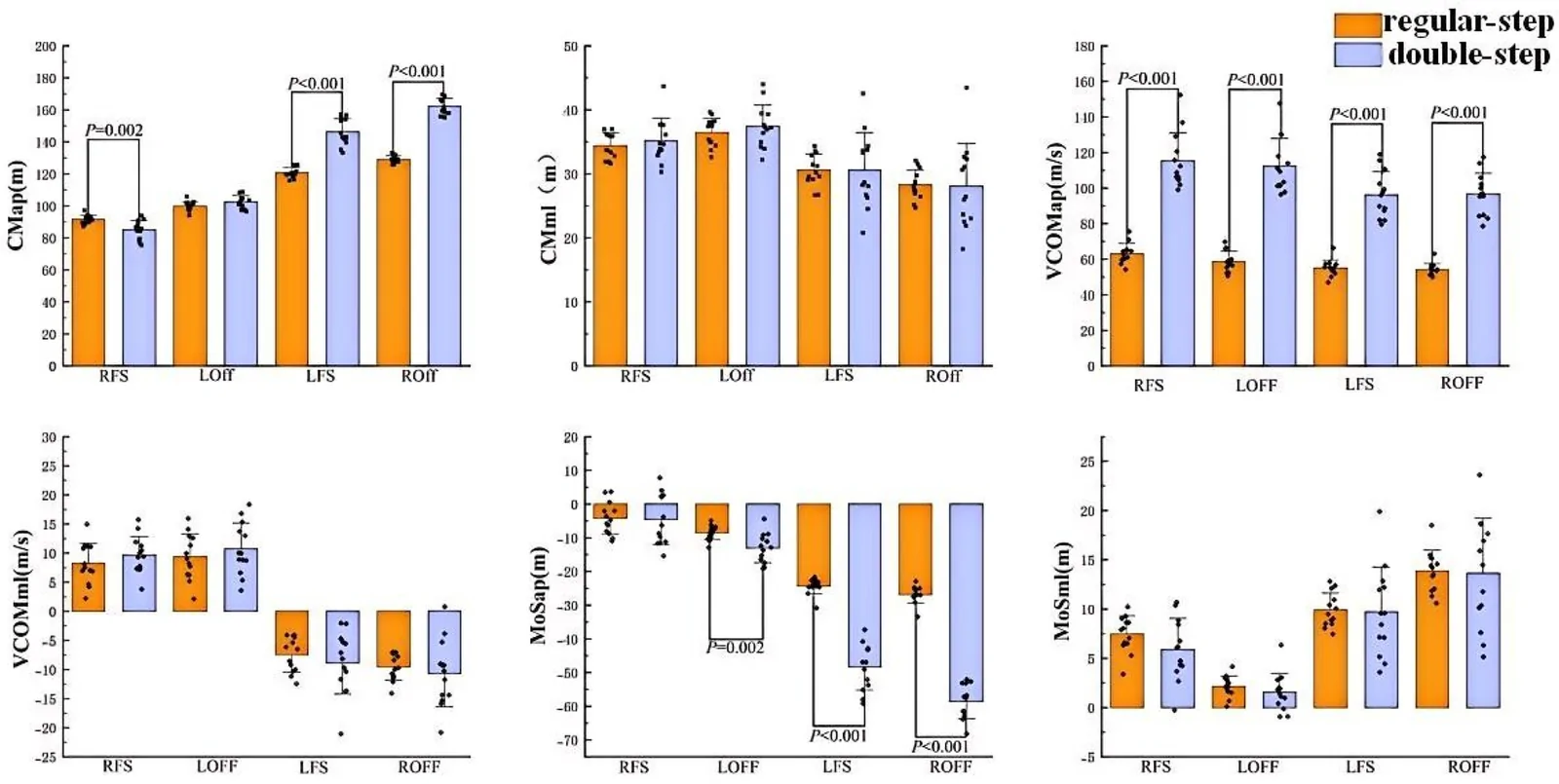
Comparison of dynamic stability between regular step-by-step and double-step strategies. MOSap represents the dynamic stability in the anterior-posterior direction, while MOSml represents that in the medial-lateral direction; CMap represents the extrapolated center of mass in the anterior-posterior direction, and CMml represents the extrapolated center of mass in the medial-lateral direction; VCOMap represents the anterior-posterior center of mass velocity; VCOMml represents the medial-lateral center of mass velocity.

Anterior-posterior COM velocity (VCOMap-indicating whole-body anterior-posterior fluctuation). The double-step strategy consistently produced a significantly larger VCOMap at all observed instants—Right Foot Strike (RFS), Left Foot Off (LOFF), Left Foot Strike (LFS), and Right Foot Off (ROFF)—with *P* < 0.001 for each comparison (Table 3). This sustained elevation in vertical center-of-mass oscillation indicates that the cross-step strategy impairs the body's ability to smoothly absorb and transfer vertical ground reaction forces. Clinically, this increased vertical chatter over repetitive stair climbs may accelerate passive joint loading and contribute to earlier muscular fatigue.

**Table 3:** Comparison of dynamic stability between regular step-by-step mode and double-step mode.

Stage	Label	Regular-Step	Double-Step	MD (95% CI)	t	*P*	Cohen's d
RFS	CMap	91.4 ± 2.7	85.0 ± 5.9	−6.4 (2.6，10.1)	4.093	0.002	1.135
	CMml	34.3 ± 2.0	35.2 ± 3.4	0.9(−3.1，1.4)	−0.893	0.389	−0.248
	VCOMap	63.1 ± 5.5	115.2 ± 15.6	−52.1(−61.6，−42.6)	−12.505	0.002	7.41
	VCOMml	8.2 ± 3.5	9.6 ± 3.1	−1.4(−4.1，1.2)	−1.062	0.312	−0.294
	MOSap	−4.2 ± 4.8	−4.5 ± 7.5	0.3(−4.7，5.4)	0.234	0.882	0.064
	MOSml	7.4 ± 1.8	5.9 ± 3.1	1.5(−0.5，3.6)	1.917	0.079	0.532
LOFF	CMap	99.6 ± 2.9	102.3 ± 3.9	−2.7(−5.4，0.1)	−2.176	0.052	−0.603
	CMml	36.4 ± 2.2	37.4 ± 3.3	1.0(−3.2，1.3)	−1.133	0.293	−0.305
	VCOMap	58.5 ± 5.7	112.4 ± 15.3	−53.9(−63.2，−44.4)	−12.711	0.001	6.323
	VCOMml	9.3 ± 3.9	10.7 ± 4.4	−1.4(−4.7，2.0)	−0.813	0.541	−0.225
	MOSap	−8.4 ± 2.1	−13.0 ± 4.3	4.6 (1.7，7.3)	3.967	0.004	1.100
	MOSml	2.1 ± 1.0	1.5 ± 1.9	0.6(−0.6，1.8)	1.061	0.314	0.294
LFS	CMap	120.7 ± 3.2	146.1 ± 8.2	−25.4(−30.4，−20.3)	−10.375	0.001	−2.999
	CMml	30.6 ± 2.4	30.6 ± 5.8	0(−3.6，3.6)	−0.006	0.994	−0.002
	VCOMap	55.0 ± 4.5	96.1 ± 12.9	−41.1(−48.9，−33.2)	−10.794	0.001	10.570
	VCOMml	−7.4 ± 3.0	−8.8 ± 5.3	1.4(−2.0，4.9)	0.847	0.479	0.265
	MOSap	−24.3 ± 2.2	−48.3 ± 6.8	24 (19.7，28.0)	11.904	0.001	3.496
	MOSml	9.8 ± 1.7	9.7 ± 4.5	0.1(−2.6，2.9)	0.137	0.961	0.040
ROFF	CMap	128.7 ± 2.4	162.3 ± 4.8	−33.6(−36.3，−30.8)	−26.732	0.001	−7.414
	CMml	28.2 ± 2.3	28.0 ± 6.6	0.2(−3.9，4.0)	0.115	0.913	0.032
	VCOMap	53.9 ± 3.5	96.5 ± 11.7	−42.6(−49.1，−35.9)	−14.02	0.001	12.002
	VCOMml	−9.5 ± 2.2	−10.6 ± 5.7	1.1(−2.5，4.7)	0.683	0.598	0.189
	MOSap	−26.8 ± 2.5	−58.6 ± 5.0	31.8 (29.1，34.4)	26.21	0.001	7.269
	MOSml	13.8 ± 2.1	13.6 ± 5.5	0.2(−3.1，3.6)	0.146	0.961	0.041

CMap – reflecting the anterior-posterior position of the CoM, which indicates the forward balance reserve and the control of whole-body COM.The double-step strategy exhibited a phase-dependent biphasic response. At RFS, CMap was significantly smaller (*P* = 0.002,), suggesting a relatively rigid, poorly modulated heel-strike pattern with reduced mediolateral damping upon initial ground contact. Conversely, at LFS and ROFF, CMap became significantly larger (both *P* < 0.001), indicating excessive center-of-pressure displacement during the subsequent single-leg support and push-off phases. This overshoot may reflect proximal muscle weakness or poor hip-knee coordination, potentially predisposing the individual to lateral instability.

Forward balance reserve (MOSap-indicating anterior-posterior stability decline).

The double-step demonstrated a significantly reduced MOSap during the majority of the stance phase, specifically at LOFF (*P* = 0.002), LFS (*P* < 0.001), and ROFF (*P* < 0.001). A smaller sagittal-plane margin of stability directly translates to a narrower safety buffer against forward center-of-mass displacement. Clinically, this consistent reduction across the weight-transfer windows (mid-stance to push-off) suggests a diminished anterior stability margin, particularly when the contralateral limb is airborne-a critical phase for community-dwelling stair climbers.

## 4 Discussion

### 4.1 Differences in dynamic stability between regular step-by-step and double-step strategies

Compared with the regular step-by-step pattern, the double-step strategy during stair ascent was associated with a significantly smaller (i.e., more negative) MOSap, reflecting a reduced anterior-posterior stability margin. In contrast, no significant differences were observed MOSml or its associated determinants, suggesting that the double-step pattern did not elevate lateral decline in stability.

Throughout the gait cycle, the MOSap values for both patterns remained negative and exhibited a progressive decline over time. At the instant of right foot strike, the MOSap was already in an unstable state and gradually transitioned towards a critically unstable condition. The significantly lower MOSap observed in the double-step condition may be attributable to the increased difficulty in controlling COM velocity and position, which compromised anterior-posterior dynamic stability.

According to the inverted pendulum model, the MOS is defined as the difference between the COP-defined functional stability boundary (BCOP) and the extrapolated centre of mass position (X > CoM) [see (7, 16)]. The position and velocity of the COM are decisive factors in determining XCoM [see (16)]. A positive MOS indicates that XCoM lies within the BOS, reflecting a stable state; conversely, a negative MOS signifies that XCoM exceeds BCOP, indicating instability. Hamel et al [see (17)] reported that increased COM velocity (VCOM) is detrimental to controlling forward displacement of the XCoM, making it difficult to maintain the XCoM within the BOS and thereby exacerbating forward stability decline. Consistent with this notion, the present study observed a larger anterior-posterior COM velocity (VCOMap) in the double-step condition. Given that XCoMis a critical determinant of MOS during stair negotiation, a greater VCOMap would inevitably increase XCoM, consequently reducing the MOSap, i.e., narrowing the anterior-posterior stability margin. This finding aligns with previous investigations [see (17)].

### 4.2 Effects of regular step-by-step and double-step strategies on patellofemoral joint biomechanics

During stair ascent, the instant of left foot off serves as a braking phase to resist the downward fall of COM. This phase generates substantial ground reaction forces, braking impulses, and knee extensor moments (KM) [see (11)]. Based on the calculation method of PFJS used in this study, this consequently results in a higher PFJS [see (14)]. At the LOFF instant, body support is primarily provided by the right leg. Furthermore, the right knee exhibits a large flexion angle in the sagittal plane at this point, necessitating greater knee flexion and Quadriceps Femoris (QF) activation to maintain body stability. Therefore, a larger PFJS is also generated at this instant [see (8, 20)].

Lower limb joint moments serve not only as biomechanical indicators but also as neural control signals, ultimately reflecting the desired control of the central nervous system (CNS). They represent characteristics of neuromuscular control and are considered one of the primary internal factors influencing postural control ability [see (11)]. In the context of stair negotiation, the extension moments of the hip and knee joints, along with the plantarflexion moment of the ankle joint, constitute the core components of the Lower Limb Support Moment. Particularly during stair ascent, the large support moment generated by the lower limbs plays a dual critical role [see (18)]: on one hand, it precisely regulates body posture and ensures dynamic stability; on the other hand, it effectively counteracts the adduction moments induced by trunk and swing leg oscillations. Therefore, the lower limb support moment is a crucial biomechanical factor stability decline during stair ascent.

Studies have found that during stair ascent, as the body accelerates forward and upward, the lower limbs require robust support, with knee extension and ankle plantarflexion moments being the primary contributors to the lower limb support moment [see (11)]. The results of this study show that the right knee joint moment peaks at the LOFF instant. At this moment, the right knee joint moment under the double-step strategy is significantly increased compared to the regular step-by-step strategy, which may theoretically provide greater support and thus could contribute to mitigating stability decline. Nevertheless, because our MOSap results indicated decreased anterior-posterior stability under the double-step condition, whether this increased knee moment actually translates into a less stability decline remains uncertain and should be investigated further. Research by Duncan et al [(see (18)] indicates that stair negotiation leads to high peak patellofemoral joint forces, which is a major cause of patellofemoral pain (PFP). Our results show that patellofemoral joint force increases significantly at the LOFF instant, and that the double-step strategy subjects the patellofemoral joint to greater forces compared with the regular strategy. In the healthy young male sample examined here, this increased load theoretically adds to the mechanical burden on the patellofemoral region. We hypothesize that, if this mechanical change were repeated over the long term, it could be associated with an elevated risk of patellofemoral pain; however, this hypothesis requires longitudinal validation and cannot be inferred from the present biomechanical data alone. It must be emphasised that, because our study did not include individuals at high risk of falls or those with diagnosed patellofemoral joint disorders, a direct causal link between the above mechanical changes and clinical symptoms cannot be established. Therefore, the current evidence is insufficient to support any definitive gait recommendations for clinical populations. We cautiously suggest that, in healthy young men, adopting a relatively cautious stepping strategy during stair ascent may help, at a mechanical level, to reduce the patellofemoral stress peak at that specific instant. However, this suggestion should be regarded only as a preliminary biomechanical hypothesis, rather than a conclusion directly derivable from the present study. Any association between the observed mechanical changes and long-term outcomes such as muscle fatigue, cartilage wear, or future patellofemoral pain remains speculative and requires further validation through interventional studies specifically targeting high-risk populations. The actual protective efficacy and clinical translational value of this strategy are not yet established.

### 4.3 Limitations

This study has several limitations. First, while the cadence was strictly controlled by a metronome, the actual forward velocity was not experimentally matched between the two gait patterns, as it is intrinsically coupled with step length and vertical displacement. Therefore, we cannot completely rule out the possibility that subtle differences in forward velocity may have partially contributed to the observed kinetic variations. Second, the analysis of dynamic stability was limited to the anterior-posterior (AP) direction at the moment of heel strike. Future studies should examine stability in both AP and mediolateral directions across the entire gait cycle. Third, all recruited participants were healthy males; other variables such as sex, age, and health status were not controlled. Future studies should explore the influence of these demographic factors on stair-ascending gait. Fourth, the testing order was not randomised; the regular step-by-step condition was always performed before the double-step condition. Although this order was chosen primarily for safety and familiarisation, it may have introduced order effects such as learning, fatigue, or habituation, which could confound the comparison between conditions. We acknowledge that we cannot disentangle these effects from the true biomechanical differences, and therefore the interpretation of condition-related outcomes should be considered with this limitation in mind. Future studies should adopt a randomised or counterbalanced design to mitigate this issue. Fifth, the tests were conducted solely in a standardised laboratory environment, and external environmental factors may have had some influence on the results. Finally, the patellofemoral biomechanical characteristics were derived from theoretical mechanical equations, which may deviate from actual *in vivo* physiological loading. Future research could employ more advanced and accurate measurement techniques to validate these findings.

## 5 Conclusion

This study confirms that the double-step (skip-step) pattern during stair ascent exhibits a dynamic stability disadvantage in the anterior-posterior direction, reflected in negative and continuously declining MOSap values. This pattern is also associated with significantly increased lower-limb support forces and a sharp rise in PFJS. However, it should be emphasized that these relationships represent observed associations rather than established causal mechanisms: our data do not allow conclusions regarding the causal direction between stability compensation and joint loading. Therefore, the coexistence of increased support moments and elevated PFJS is more appropriately characterized as a potential trade-off between “stability compensation” and “joint overload” that warrants further investigation, rather than as a confirmed mechanism. Given that no clear risks were identified in the mediolateral direction, these findings may have relevance for gait guidance. It is important to note, however, that our study did not directly investigate older adults, individuals with balance impairments, or patients with patellofemoral pain. Consequently, any specific recommendations for these populations should be regarded as hypotheses or future research directions, rather than as clinical recommendations at the current stage. Further studies are needed to assess the applicability of these strategies and to explore how an optimal balance between safety and joint protection might be achieved in these specific groups.

## Data Availability

The original contributions presented in the study are included in the article/Supplementary Material, further inquiries can be directed to the corresponding author.
